# Identifying Populations at High Risk for Diabetes With the Behavioral Risk Factor Surveillance System, Rhode Island, 2003

**Published:** 2010-06-15

**Authors:** Kathryn Wojcik, Annie Gjelsvik, Dona Goldman

**Affiliations:** Warren Alpert Medical School, Brown University, Providence, Rhode Island. Ms Wojcik is also affiliated with Coastal Medical, Providence, Rhode Island; Brown University. Dr Gjelsvik is also affiliated with the Rhode Island Department of Health Diabetes Prevention and Control Program.; Rhode Island Department of Health Diabetes Prevention and Control Program, Providence, Rhode Island

## Abstract

**Introduction:**

We evaluated the feasibility of applying a previously validated diabetes risk score (DRS) to state-based surveillance data from the Behavioral Risk Factor Surveillance System (BRFSS) to assess population risk for developing type 2 diabetes or having undiagnosed type 2 diabetes.

**Methods:**

We conducted a cross-sectional analysis of 1,969 adults aged 30 to 60 years who self-reported never having been diagnosed with diabetes. The Danish DRS was applied to the 2003 Rhode Island BRFSS data by using 6 categorical variables: age, sex, body mass index, known hypertension, leisure-time physical activity, and family history of diabetes. The DRS was the sum of these individual scores, which ranged from 0 to 60; a score of 31 or more was considered high-risk.

**Results:**

We found that 436 study participants, representing 23% of Rhode Island adults aged 30 to 60 years, had a high DRS. In the final model, adults with at least some college education were 43% less likely to have a high DRS, compared to adults with a high school diploma. Adults with no health insurance were 54% more likely to have a high DRS compared with insured adults.

**Conclusion:**

By adding a family history question in odd years to correspond to the hypertension module in the BRFSS, routinely available state-level surveys can be used with a DRS to monitor populations at high risk for developing type 2 diabetes. In Rhode Island, almost one-fourth of adults aged 30 to 60 years were at high risk for having undiagnosed diabetes or developing diabetes. Adults with lower education and without health insurance were at highest risk.

## Introduction

Type 2 diabetes often goes undetected for many years. When patients are diagnosed with diabetes, 25% have established retinopathy, and half have clinical evidence of diabetic tissue damage. These measurements have been extrapolated to estimate a delay from disease onset to diagnosis of more than 10 years (1). Moreover, type 2 diabetes can be prevented or delayed in people with impaired glucose tolerance (IGT) with lifestyle modification or prescription drug treatment (2). A major task for public health professionals is to identify people at high risk for developing diabetes who would benefit from these interventions.

Researchers have created diabetes risk score (DRS) tools based on data that are routinely available in primary care and have validated them in many populations (3-12). For a study conducted in Denmark, researchers developed a simple self-administered questionnaire to identify people with undiagnosed diabetes and derived a DRS from it. The Danish DRS is useful for identifying people who have undiagnosed diabetes or are at high risk for developing diabetes. In addition, the Danish DRS study concluded that the use of this tool may decrease the number of subsequent tests and potentially could lower screening costs (8).

In addition to clinical applications, a DRS could prove useful when applied to surveillance data to assess population risk of developing diabetes. This information can help target high-risk populations that clinical application may miss, such as people without health insurance. In addition, screening for diabetes may be more efficient if targeted at high-risk populations (13). DRS questionnaires have mainly been used in primary care because they are less labor-intensive and more acceptable to patients than biochemical screening tests such as measurement of fasting glucose or glycosylated hemoglobin (1).

Projection of disease prevalence helps in planning for health care needs. Studies have projected diagnosed diabetes prevalence for the United States and other countries (14,15). By understanding the proportion and distribution of adults at high risk for developing diabetes or having undiagnosed diabetes, more accurate estimates of prevalence can be calculated and prevention activities can be better targeted.

Our objective was to evaluate the feasibility of using a validated DRS with state-based surveillance data from the Behavioral Risk Factor Surveillance System (BRFSS) to assess population risk for developing diabetes and to identify populations for screening and targeting interventions.

## Methods

We used data from the 2003 Rhode Island BRFSS for this analysis. The BRFSS is a standardized, telephone survey of noninstitutionalized adults aged 18 years or older in the United States, Guam, Puerto Rico, and the Virgin Islands (16). The standard BRFSS questionnaire has 3 components: the core, optional modules, and state-added questions. The core questionnaire is a set of standardized questions asked of all respondents in each state and territory. Optional modules are sets of standardized questions that each state can select about specific topics such as diabetes and arthritis management. State-added questions are designed and used by states to address their specific needs.

### The Danish Diabetes Risk Score

We used the Danish DRS for our research. The Danish DRS was developed with half of the 6,124 eligible participants of the Inter99 study, validated internally with the other half of the Inter99 participants and validated externally with 1,028 ADDITION pilot study participants (8). Frequent thirst, frequent voluminous voiding of urine, weight loss, tiredness, repeated cystitis, age, sex, body mass index (BMI), family history of diabetes (parents or siblings), known hypertension, antihypertensive treatment, knowledge of dyslipidemia, treatment for hypercholesterolemia, and leisure-time physical activity were considered for this DRS. Three steps were used to select variables in the final Danish DRS. If diabetes was strongly associated with the variable then it was next evaluated by using logistic regression, with screen-detected diabetes as the outcome. Participants were considered to have screen-detected diabetes if they did not know they had diabetes but had a fasting plasma glucose level ≥7.0 mmol/L or 2-hour plasma glucose level ≥11.1 mmol/L. Variables with a *P* value of <.20 from this model were included in a stepwise backward elimination process, with screen-detected diabetes as the dependent variable, resulting in 6 variables (age, sex, BMI, known hypertension, leisure-time physical activity, and parental diabetes) in the final model. The score was constructed by multiplying the regression coefficient by 10 and rounding to the nearest integer (Table 1). The sum of each variable score was calculated for each participant, resulting in a DRS ranging from 0 to 60. Because a score of 31 or more showed a sensitivity close to the prespecified value of 75%, this value was chosen as the cutoff for high risk and was the only cutoff that was validated (8).

### Study sample

We selected the 2003 Rhode Island BRFSS because this was the only year that included all 6 of the variables in the Danish DRS. The study sample included Rhode Island residents aged 30 to 60 years who said they had not been told by a doctor that they have diabetes and who had valid responses for all items included in the Danish DRS and all potential predictor variables (n = 1,969). Respondents were identified as having diabetes if they answered yes to the question, "Have you ever been told by a doctor that you have diabetes?" (17). Respondents who answered "do not know/not sure" or "refused" and women who had diabetes only during pregnancy were classified as not having diabetes and were included in the study sample.

### Study variables

We divided age into 4 categories: 30 to 40, 41 to 47, 48 to 54, and 55 to 60. Sex was a binary category. BMI was divided into 3 categories: <25.0 kg/m^2^, 25.0-29.9 kg/m^2^, and ≥30 kg/m^2^. The BRFSS question used to define known hypertension was, "Have you ever been told by a doctor, nurse, or other health professional that you have high blood pressure?" (17). Respondents who answered yes were classified as having hypertension and those who answered no or only during pregnancy were classified as not having hypertension (Table 1).

In the Danish DRS, leisure-time physical activity was a binary (yes/no) variable (8). BRFSS respondents were asked, "During the past month, other than your regular job, did you participate in any physical activities or exercises such as running, calisthenics, golf, gardening, or walking for exercise?" (17) (Table 1).

The final variable in the Danish DRS was whether the respondent had a parent with diabetes. The 2003 Rhode Island BRFSS contains a state-added question related to family history of diabetes: "This question asks about your family members who are related to you by blood (do not include diabetes during pregnancy). Do you have a parent, brother, or sister related by blood, who has or has had diabetes?" (17). The response was categorized into a binary (yes/no) variable. Respondents who answered "not sure/do not know" or "refused" to any of these variables were not included in the analyses.

We evaluated potential predictors that have been demonstrated in previous studies to be related to undiagnosed diabetes or high diabetes risk that were not already included in the DRS: race/ethnicity (18), education (19), employment (20), health insurance (21), and smoking status (1). In addition, we evaluated marital status and having an annual checkup as potential predictors.

### Statistical analyses

To account for the complex survey design, we conducted all analyses by using Stata version 10.0 (StataCorp LP, College Station, Texas). First we calculated frequencies for potential predictors. Then we calculated odds ratios (ORs) and 95% confidence intervals (CIs) for each potential predictor. Our third step was to run a full model that included all potential predictors. Our final model included race/ethnicity, education, employment, health insurance, and smoking status, based on previous literature (1,18-21), and added annual checkup based on the Wald test. We assessed this model by using a goodness-of-fit statistic developed for complex survey design (22).

## Results

Approximately 23% of adults in Rhode Island aged 30 to 60 years who had not been diagnosed with diabetes had a high risk of developing diabetes according their Danish DRS category. Compared to respondents with a low DRS, a greater percentage of those with a high DRS had less than a high school education, were retired, had no health insurance, and had seen a doctor within the past 12 months (Table 2).

Crude estimates showed that adults who had less than a high school degree or General Educational Development certificate (GED) were 58% more likely and those with at least some college were 39% less likely to have a high Danish DRS compared to people with a high school degree or GED. In addition, respondents who were unemployed were 51% more likely and those who were retired were more than 5 times as likely to have a high Danish DRS as adults who were employed (Table 3).

The final model contained race/ethnicity, education, employment, health insurance, smoking status, and annual checkup. It showed no evidence of lack of fit (*F* = 0.80, *P* = .61). In the final model, adults with at least some college, current smokers, and those who had not seen a doctor in the past 12 months had lower odds of a high DRS, and retired adults and adults without health insurance had higher odds of a high DRS. There was no association between race/ethnicity and high DRS (Table 3).

## Discussion

Based on 2003 Rhode Island BRFSS data, 7% of Rhode Island adults had diagnosed diabetes, which corresponds to national estimates (23), and we found that 23% of Rhode Island adults aged 30 to 60 years without previous diagnosis of nongestational diabetes are at high risk for having undiagnosed diabetes or developing diabetes. This estimate is consistent with estimates of undiagnosed diabetes and diabetes risk from National Health and Nutrition Examination Survey (NHANES) data. Using 1999-2002 NHANES data, Cowie and colleagues estimated that 28.8% of adults aged 20 or older had either undiagnosed diabetes (2.8%) or impaired fasting glucose (IFG) (26.0%) (24). These estimates varied by age; 33.2% of 40- to 59-year-olds and 16.6% of 20- to 39-year-olds have either undiagnosed diabetes or IFG (24). When incorporating IGT into the definition of high-risk so that people were identified as having prediabetes if they had IFG or IGT, Cowie and colleagues estimated that 29.5% of adults aged 20 or older were at high risk for diabetes on the basis of 2005-2006 NHANES data (25).

We found that education and health insurance status were associated with diabetes risk, which is consistent with previous research (19,21). In the final model, adults with at least some college education were 43% less likely to have a high DRS than were adults with a high school education, and adults without health insurance were 54% more likely to have a high DRS than were adults with health insurance. Retired adults had 5.43 the odds of a high DRS compared with employed adults, which is likely due to the increased risk of diabetes associated with aging.

Current smokers were 30% less likely to have a high DRS compared with adults who did not smoke. This finding may be due to the differences in BMI between smokers and nonsmokers (26). In our sample, 48% of smokers were neither overweight nor obese compared with 39% of nonsmokers. Contrary to what we expected to find, adults who had not seen a doctor in the past year were 39% less likely to have a high DRS compared with adults who had seen a doctor in the past year. This could be due to the way hypertension was assessed. Participants needed to have been told by a doctor, nurse, or other health professional that they had hypertension to be categorized accordingly; thus, adults without regular checkups may be less likely to have a high DRS because of undiagnosed hypertension. In addition, other variables that contribute to a high DRS such as increasing age, family history of diabetes, and obesity may increase the likelihood of seeing a doctor regularly.

Unlike previous research (27), we did not find any differences in diabetes risk by race/ethnicity. One reason is that the Danish DRS was developed only for white populations (8) and does not take race/ethnicity into account as other DRS tools do (10-12). We ran our final model using only non-Hispanic white adults and the results were essentially the same: lower education, having a health plan, not smoking, and having an annual checkup were associated with a higher DRS. The increased odds of high DRS for unemployed adults compared with employed adults became significant (OR, 1.42; 95% CI, 1.00-2.01).

Our study had several limitations. First, the Danish DRS was developed and validated in different populations than our study population. Risk scores that have been developed and validated in 1 population may not have adequate sensitivity or specificity in other populations (28). Although the Rhode Island population is primarily non-Hispanic white (29), there are likely differences between the European and Rhode Island populations on DRS items. For instance, the Rhode Island study population had higher rates of obesity, known hypertension, and reported leisure-time physical activity compared with the Inter99 and ADDITION pilot study populations (8).

Second, although we tried to duplicate the Danish DRS as closely as possible, we were limited to the population and items available on the Rhode Island BRFSS, since we were primarily interested in determining and monitoring high diabetes risk in a state population. The BRFSS is a telephone survey of noninstitutionalized adults; thus, we did not have information on adults in prisons, long-term care facilities, or without landline telephones. These populations may have a higher risk of diabetes (30). Additionally, all information on the BRFSS is self-reported, in contrast to the pilot study of the Inter99 and ADDITION populations, in which blood pressure, height, and weight were measured (8). Women tend to underestimate their weight and men to overestimate their height (31), which would result in an underestimate, in the Rhode Island population, of diabetes risk.

There were also differences in the questions used to assess family history of diabetes and leisure-time physical activity. In the Inter99 study, family history of diabetes was assessed in 2 parts: parental and sibling. The Danish DRS included only parental history of diabetes, although the ADDITION pilot study, 1 of the populations in which the Danish DRS was validated, used only 1 question. This was similar to the Rhode Island BRFSS, which combined parental and sibling history of diabetes. In addition, because the family history question used in the Rhode Island BRFSS was state-added it has not, to our knowledge, been validated. This study may therefore overestimate the prevalence of high diabetes risk.

We used a single question on reported leisure-time physical activity to categorize participants as active or inactive, whereas the Danish DRS used 4 original categories (sedentary, moderate, active, and competitive sport), which were collapsed into active vs inactive. Differences in how leisure-time physical activity was measured could result in inaccurate estimation of population diabetes risk. We therefore explored 2 other ways of categorizing adults using the BRFSS data. The first used information on any activity vs no moderate or vigorous activity. The second used information on days and minutes of moderate and vigorous physical activity and adhered to the *Healthy People 2010* definition of active and inactive (32). With all 3 ways of categorizing physical activity we found similar prevalence of high diabetes risk (23.1%, 23.8%, and 22.1%) and similar relationships with potential predictors. Thus, we used the 1-question original method, since this resulted in the fewest observations discarded because of missing information.

We would have preferred a DRS that had been validated in a US population with similar demographics to Rhode Island and that used the same questions as the Rhode Island BRFSS. We were unable to find another validated DRS that could be applied to state-level surveillance data because the BRFSS does not ask participants their waist circumference, which is a common variable in other validated DRS questionnaires (3-12).

Data collected routinely in the BRFSS can identify populations at risk of diabetes more efficiently than complicated and costly screening strategies. Our method of using the Danish DRS applied to the BRFSS offers a new way for states to determine and target the population at risk for diabetes. In addition, because the BRFSS is a routine surveillance system for states, states can track high-risk populations over time. A state-added question about family history of diabetes should be included in the same year as the rotating hypertension awareness module.

This study indicates that approximately 23% of the Rhode Island adult population is at high risk for diabetes and that particular risk groups such as people with less education and without health insurance should be targeted for diabetes prevention and screening.

## Figures and Tables

**Table 1:** Characteristics Used to Calculate Danish Diabetes Risk Score^a^

Characteristic	Assigned Score
**Age, y**	
30-40	0
41-47	7
48-54	13
55-60	18
**Sex**	
Men	4
Women	0
**Body mass index, kg/m^2^**	
<25.0	0
25.0-29.9	7
≥30.0	15
**Known hypertension**	
Yes	10
No	0
**Leisure-time physical activity**	
Yes	0
No	6
**Family history of diabetes**	
Yes	7
No	0

a Glümer et al (8).

**Table 2:** Diabetes Risk by Selected Characteristics of Respondents to the Rhode Island Behavioral Risk Factor Surveillance System (N = 1,969), 2003

Characteristic	No. of Low-Risk Respondents^a^ (%) n = 1,533	No. of High-Risk Respondents^a^ (%) n = 436	Total No. of Respondents (%)
**Race/ethnicity**			
White, non-Hispanic	1,328 (87.5)	373 (86.7)	1,701 (87.3)
Black, non-Hispanic	42 (2.4)	12 (2.8)	54 (2.5)
Other/multirace, non-Hispanic^b^	57 (4.2)	11 (2.2)	68 (3.7)
Hispanic	106 (5.9)	40 (8.3)	146 (6.5)
**Education**			
Less than high school	84 (5.1)	47 (10.7)	131 (6.4)
High school graduate/GED	348 (24.2)	140 (32.0)	488 (26.0)
Some college	1,101 (70.8)	249 (57.3)	1,350 (67.6)
**Employment^c^**			
Employed	1,232 (81.5)	297 (71.2)	1,529 (79.1)
Unemployed	276 (17.2)	114 (22.7)	390 (18.4)
Retired	25 (1.4)	25 (6.2)	50 (2.5)
**Marital status**			
Married	865 (66.9)	234 (67.3)	1,099 (67.0)
Unmarried^d^	668 (33.1)	202 (32.7)	870 (33.0)
**Health insurance coverage**			
Yes	1,404 (92.2)	380 (88.5)	1,784 (91.3)
No	129 (7.8)	56 (11.5)	185 (8.7)
**Smoking status**			
Smoker^e^	383 (24.7)	98 (20.7)	481 (23.8)
Nonsmoker	1,150 (75.3)	338 (79.3)	1,488 (76.2)
**Annual checkup^f^**			
Yes	1,211 (78.9)	370 (85.4)	1,581 (80.4)
No	322 (21.1)	66 (14.6)	388 (19.6)

Abbreviation: GED, General Educational Development certificate.

a As determined by the Danish Diabetes Risk Score (8), 0-30 is low-risk and 31-60 is high-risk.

b Other includes Asian, Native Hawaiian/Other Pacific Islander, American Indian/Alaska Native, other, and multiracial

c Employed includes respondents who self-reported employed for wages and self-employed. Unemployed includes homemakers and students.

d Unmarried includes divorced, widowed, separated, never married, or member of an unmarried couple

e Current smoker includes respondents who self-reported smoking cigarettes every day or some days.

f Annual checkup refers to checkup within the last year.

**Table 3:** Odds of Having a High Danish Diabetes Risk Score, by Selected Characteristics of Respondents to the Rhode Island Behavioral Risk Factor Surveillance System, 2003

Characteristic	Crude OR (95% CI)	AOR^a^ (95% CI)	Final Model^b^AOR (95% CI)
**Race/ethnicity**			
White, non-Hispanic	1 [Reference]	1 [Reference]	1 [Reference]
Black, non-Hispanic	1.21 (0.56-2.59)	1.22 (0.54-2.79)	1.21 (0.53-2.77)
Other/multirace, non-Hispanic^c^	0.52 (0.25-1.09)	0.52 (0.24-1.10)	0.52 (0.25-1.11)
Hispanic	1.42 (0.89-2.62)	0.85 (0.52-1.40)	0.85 (0.51-1.39)
**Education**			
Less than high school	1.58 (0.96-2.60)	1.56 (0.94-2.59)	1.54 (0.92-2.56)
High school graduate/GED	1 [Reference]	1 [Reference]	1 [Reference]
Some college	0.61 (0.46-0.80)	0.57 (0.42-0.77)	0.57 (0.42-0.77)
**Employment^d^**			
Employed	1 [Reference]	1 [Reference]	1 [Reference]
Unemployed	1.51 (1.21-2.04)	1.33 (0.97-1.83)	1.33 (0.97-1.82)
Retired	5.20 (2.74-9.86)	5.47 (2.85-10.47)	5.43 (2.84-10.39)
**Marital status**			
Married	1 [Reference]	1 [Reference]	NI
Unmarried^e^	0.98 (0.77-1.26)	0.91 (0.70-1.17)	NI
**Health insurance coverage**			
Yes	1 [Reference]	1 [Reference]	1 [Reference]
No	1.53 (1.02-2.30)	1.58 (1.04-2.40)	1.54 (1.01-2.33)
**Smoking status**			
Current smoker^f^	0.80 (0.59-1.08)	0.72 (0.51-1.00)	0.70 (0.50-0.98)
Nonsmoker	1 [Reference]	1 [Reference]	1 [Reference]
**Annual checkup^g^**			
Yes	1 [Reference]	1 [Reference]	1 [Reference]
No	0.64 (0.45-0.91)	0.61 (0.43-0.87)	0.61 (0.43-0.87)

Abbreviations: OR, odds ratio; CI, confidence interval; AOR, adjusted odds ratio; GED, General Educational Development certificate; NI, not included.

a Contains race/ethnicity, education, employment, marital status, health insurance status, smoking status, and annual checkup.

b Contains race/ethnicity, education, employment, health insurance status, smoking status, and annual checkup.

c Other includes Asian, Native Hawaiian/Other Pacific Islander, American Indian/Alaska Native, other, and multiracial.

d Employed includes respondents who self-reported employed for wages and self-employed. Unemployed includes homemakers and students.

e Unmarried includes divorced, widowed, separated, never married, or member of an unmarried couple.

f Current smoker includes respondents who self-reported smoking cigarettes every day or some days.

g Annual checkup refers to checkup in the last year.
